# Supplementary material for: Enhanced beta power emerges from simulated parkinsonian primary motor cortex
Source: NPJ Parkinsons Dis. 2025 Aug 5;11:230. doi: 10.1038/s41531-025-01070-4 (PMC12325672; doi:10.1038/s41531-025-01070-4)
Supplement: Supplementary file 1 — Supplementary information [file 41531_2025_1070_MOESM1_ESM.pdf]

Supplementary Data

Enhanced beta power emerges from simulated parkinsonian primary motor cortex

Donald W Doherty[1,2,*], Liqiang Chen[2,3], Yoland Smith[2,4], Thomas Wichmann[2,4], Hong-Yuan Chu[2,3], William W Lytton[1,2,5]

[1] Department of Physiology & Pharmacology, SUNY Downstate Medical Center, Brooklyn, NY 11203, USA

[2] Aligning Science Across Parkinson's (ASAP) Collaborative Research Network, Chevy Chase, MD, 20815

[3] Department of Pharmacology and Physiology, Georgetown University Medical Center, Washington D.C., USA

[4] Emory National Primate Research Center, Department of Neurology, Udall Center of Excellence for Parkinson's Disease Research, Emory University, School of Medicine, Atlanta GA 30329 USA

[5] Kings County Hospital, Brooklyn, NY 11203, USA

[*] donald.doherty@neurosim.downstate.edu

RESOURCE TYPE	RESOURCE NAME	SOURCE	IDENTIFIER	NEW/ REUSE	ADDITIONAL INFORMATION
Dataset	Simulated mouse primary motor cortex activity during control and decreased pyramidal tract neuron excitability in the parkinsonian condition (RRID:SCR_017571)	DANDI Archive website	https://dandiarchive.org/dandiset/001444	new	Four complete datasets of simulated mouse primary motor cortex for 6-OHDA experiments. Each dataset includes 4 NWB files (16 files total).
Dataset	NeuroMorpho.Org (RRID:SCR_002145)	NeuroMorpho.Org website	https://neuromorpho.org archive name Suter_Shepherd	reuse	Centrally curated inventorys of digitally reconstructed neurons and glia associated with peer-reviewed publications
Software/code	Python Programming Language version 3.9.18 (RRID:SCR_008394)	Python website	https://www.python.org/downloads/release/python-3918/	reuse	
Software/code	NEURON version 8.2.2 (RRID:SCR_005393)	NEURON website	https://www.neuron.yale.edu	reuse	
Software/code	NetPyNE version 1.0.5 (RRID:SCR_014758)	NetPyNE website	https://netpyne.org	reuse	
Software/code	Complete code for simulated mouse primary motor cortex 6-OHDA experiments	Github / Zenodo	http://doi.org/10.5281/zenodo.12399983	new	Detailed primary motor cortex simulation for observing changes in cortical dynamics due to changes in pyramidal tract neuron excitability in parkinsonian mice

Supplementary Table 1. Key Resource Table.

Experiment ID	Seeds	Condition	State	Mean PT5B firing rate (Hz)	Standard deviation	Significance (p<value)
**sM1_12-12-2023_01**	conn': 4321, 'stim': 1234, 'loc': 4321	Control	Rest	11.7	9.4	
**sM1_12-12-2023_02**	conn': 4321, 'stim': 1234, 'loc': 4321	Parkinsonian	Rest	14.2	7.1	0.001
**sM1_12-12-2023_03**	conn': 4321, 'stim': 1234, 'loc': 4321	Control	Activated	26.8	18.2	
**sM1_12-12-2023_04**	conn': 4321, 'stim': 1234, 'loc': 4321	Parkinsonian	Activated	29.7	12.7	0.001
**sM1_04-22-2024_01**	conn': 1592, 'stim': 3752, 'loc': 5368	Control	Rest	11.3	8.0	
**sM1_04-22-2024_02**	conn': 1592, 'stim': 3752, 'loc': 5368	Parkinsonian	Rest	19.1	9.5	0.001
**sM1_04-22-2024_03**	conn': 1592, 'stim': 3752, 'loc': 5368	Control	Activated	25.7	18.4	
**sM1_04-22-2024_04**	conn': 1592, 'stim': 3752, 'loc': 5368	Parkinsonian	Activated	29.1	11.7	0.001
**sM1_04-25-2024_01**	conn': 8392, 'stim': 1893, 'loc': 9272	Control	Rest	12.4	10.2	
**sM1_04-25-2024_02**	conn': 8392, 'stim': 1893, 'loc': 9272	Parkinsonian	Rest	22.8	11.9	0.001
**sM1_04-25-2024_03**	conn': 8392, 'stim': 1893, 'loc': 9272	Control	Activated	26.4	18.3	
**sM1_04-25-2024_04**	conn': 8392, 'stim': 1893, 'loc': 9272	Parkinsonian	Activated	30.1	12.5	0.001
**sM1_04-26-2024_01**	conn': 3525, 'stim': 3894, 'loc': 6942	Control	Rest	13.4	10.3	
**sM1_04-26-2024_02**	conn': 3525, 'stim': 3894, 'loc': 6942	Parkinsonian	Rest	24.6	12.2	0.001
**sM1_04-26-2024_03**	conn': 3525, 'stim': 3894, 'loc': 6942	Control	Activated	26.6	18.3	
**sM1_04-26-2024_04**	conn': 3525, 'stim': 3894, 'loc': 6942	Parkinsonian	Activated	30.7	12.4	0.001

Supplementary Table 2. Four datasets, each using different connectivity, stimulation, and neuron location random seeds. Significance (p values) are for Pyramidal Tract (PT5B) neuron firing frequency between conditions (control or parkinsonian) in a particular state (rest or activated).

Condition	BK	NaT
**Control**	7.25x10-5	0.00086
**Parkinsonian**	7.25x10-4	0.0172

Supplementary Table 3. Pyramidal Tract (PT5B) neuron parameters in control and 6-OHDA parkinsionian simulations. Ca-V-sensitive big potassium (BK), and transient sodium (NaT) channels changed Current densities in S/cm^2.

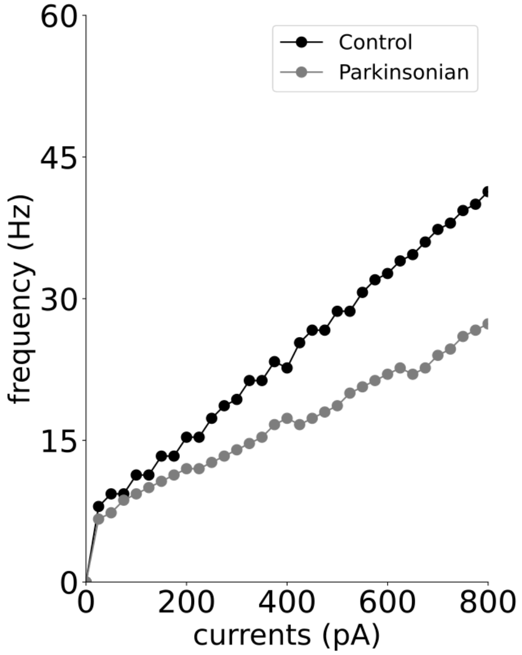

Supplementary Figure 1. Simulated Pyramidal Tract (PT5B) neuron current frequency curves for control (black) and parkinsonian (gray) conditions.

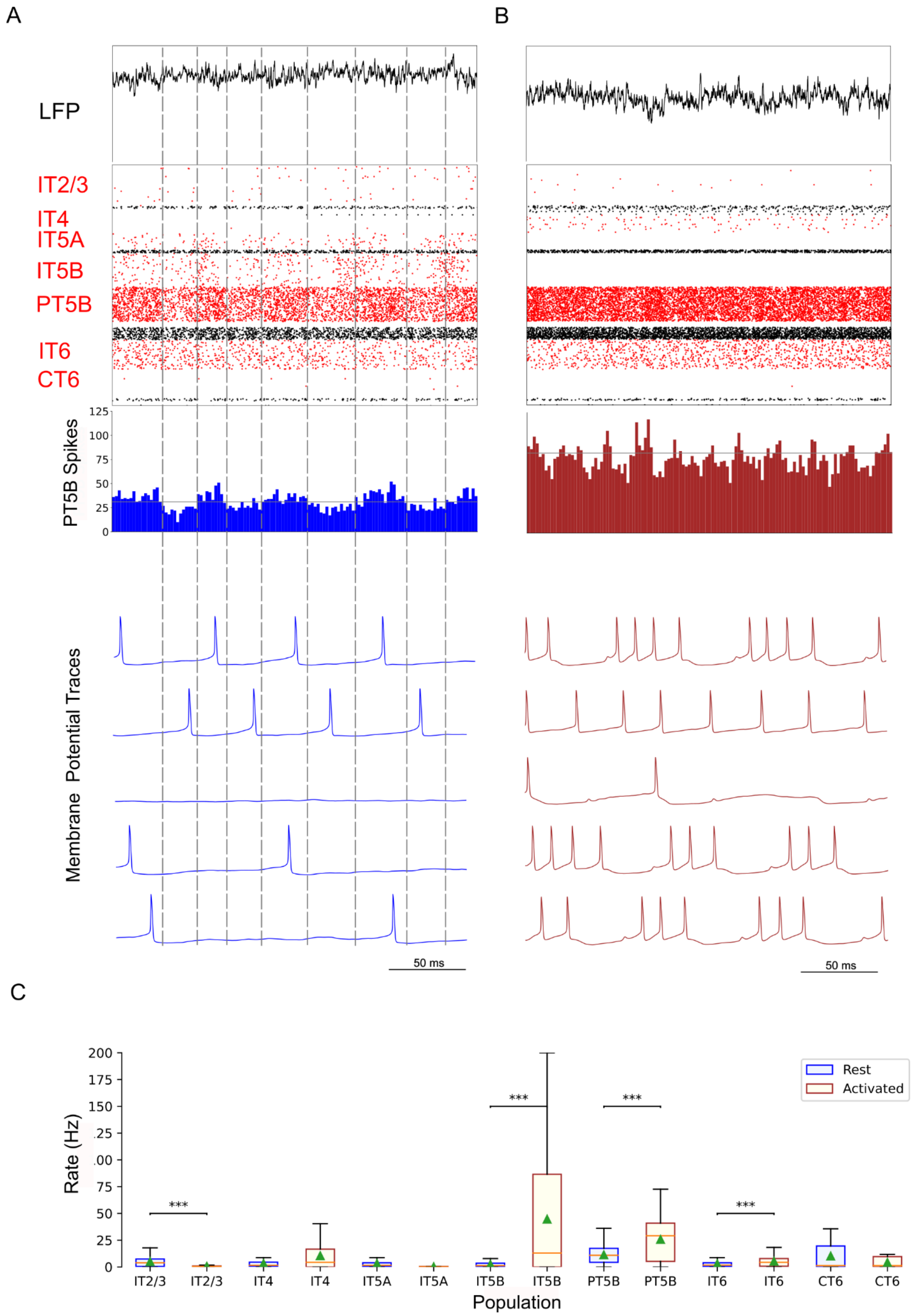

Supplementary Figure 2. Control condition, simulated primary motor cortex (M1) activity, comparing resting state to movement state. A. Resting state (250 ms at midpoint of 4.3 s simulation) displayed dominant beta-band activity (~20 Hz). Top-to-bottom: local field potential (LFP), raster plot (red excitatory; black inhibitory neurons), PT5B spike count histograms (2 ms bins; dashed lines at half height), and 5 randomly chosen PT5B voltage traces. **B.** Activated state showed change to high-frequency gamma-band activity (~44 Hz). Same top-to-bottom as

in A; bottom traces are the same neurons as in A. **C.** Statistically significant change was found for firing rates for excitatory cell types going from rest to activated: IT2/3 -- rest: 5.0±5.2 spikes/s; activated: 0.64±0.79 spikes/s; PT5B -- 11.3±8.0 vs 25.7±18.3 spikes/s; IT5B -- rest: 2.4±3.0 vs 44.6±59.3 spikes/s; IT6 neurons -- 3.0±3.8 vs 5.3±5.1 spikes/s (mean, std dev, range shown; ***p<0.001; calculated from the last 2.3 s of 4.3 s simulation to avoid initialization transients). sM1_04-22-2024.

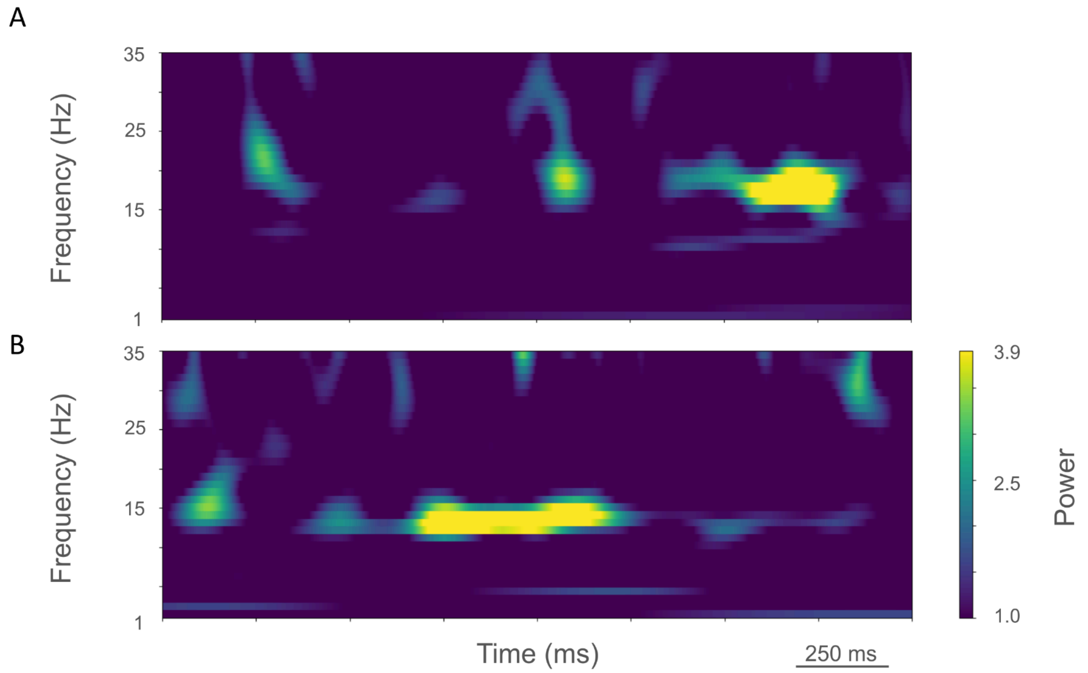

Supplementary Figure 3. Time-resolved spectrograms of cortical LFPs in M1 in the control condition, under rest and activation states. A. The rest state simulation revealed 15-25 Hz beta-band bursts. **B.** During the activated state beta bursts shifted to 15 Hz bursts and higher frequency brief bursts of activity 25-35 Hz are visible. (2.3 s of activity starting 2 s after simulation initiation. Color coding power in bar x 10^{-5}). sM1_04-22-2024.

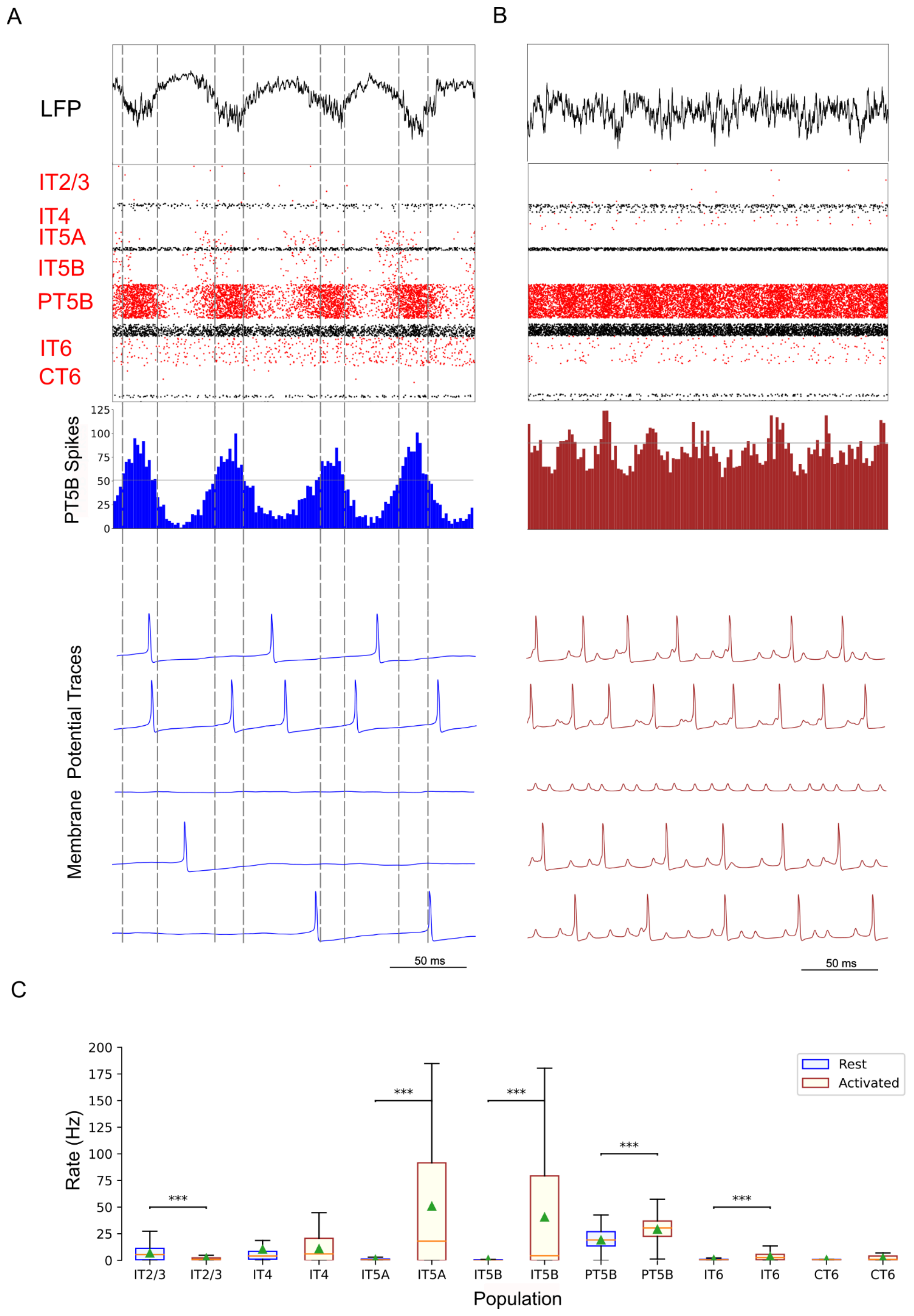

Supplementary Figure 4. Parkinsonian condition, simulated primary motor cortex (M1) activity, comparing resting state to movement state. A. Resting state (250 ms shown at midpoint of 4.3 s simulation) displayed focused 15 Hz beta-band activity. Top-to-bottom: local field potential (LFP), raster plot (red excitatory; black inhibitory neurons), PT5B spike count histograms (2 ms bins; left: dashed lines at half height), and 5 randomly chosen PT5B voltage traces. **B.** Activated state showed change to high-frequency gamma-band activity (~43 Hz).

Same top-to-bottom as in A; bottom traces are the same neurons as in A. **C.** Statistically significant change was found for firing rates for excitatory cell types going from rest to activated: IT2/3 -- rest: 6.8±6.5 spikes/s; activated: 2.1±3.4 spikes/s; IT5A -- rest: 0.89±1.2 vs 50.8±60.5 spikes/s; IT5B -- rest: 0.55±1.2 vs 40.6±55.2 spikes/s; PT5B -- rest: 19.1±9.5 vs 29.1±11.7 spikes/s; IT6 -- rest: 0.76±1.5 vs 3.6±3.8 spikes/s (mean, std dev, range; ***p<0.001; calculated from the last 2.3 s of 4.3 s simulation to avoid initialization transients). sM1_04-22-2024.

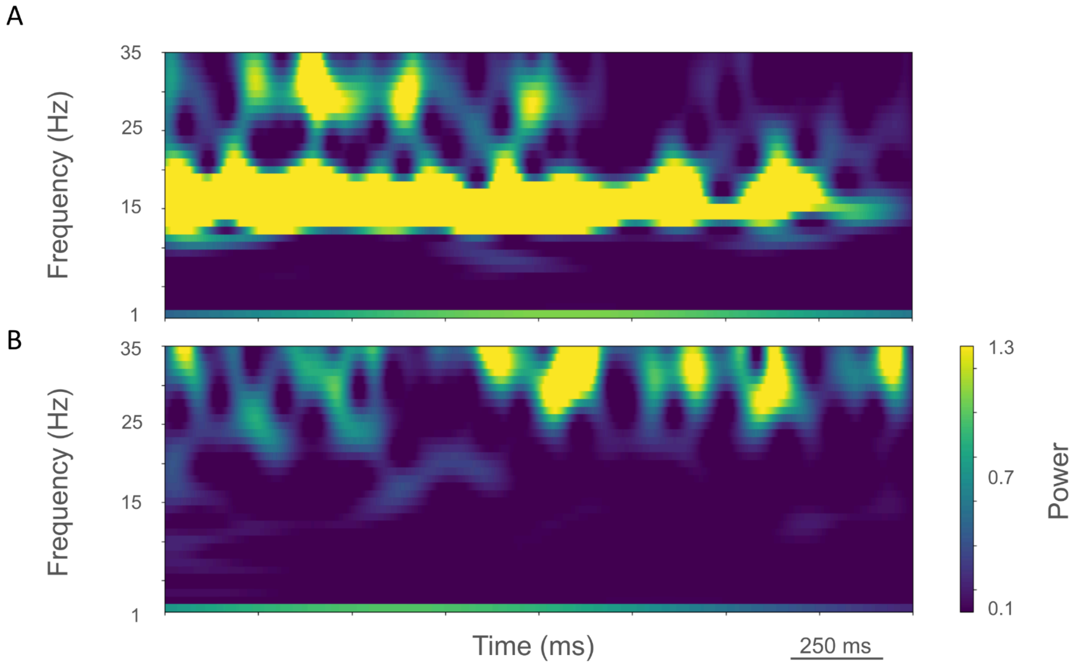

Supplementary Figure 5. Time-resolved spectrograms of cortical LFPs in M1 in the parkinsonian condition, under rest and activation states. A. The rest state simulation revealed a continuous high-power 15 Hz beta-band power with bursts of power in the ~25-35 Hz. **B.** During the activated state, 15 Hz power was no longer visible and ~20-35 Hz bursts appeared. (2.3 s of activity starting 2 s after simulation initiation. Color coding power in bar x 10^{-4}). sM1_04-22-2024.
